# Preclinical Study of Single-Dose Moxidectin, a New Oral Treatment for Scabies: Efficacy, Safety, and Pharmacokinetics Compared to Two-Dose Ivermectin in a Porcine Model

**DOI:** 10.1371/journal.pntd.0005030

**Published:** 2016-10-12

**Authors:** Charlotte Bernigaud, Fang Fang, Katja Fischer, Anne Lespine, Ludwig Serge Aho, Dominique Dreau, Andrew Kelly, Jean-François Sutra, Francis Moreau, Thomas Lilin, Françoise Botterel, Jacques Guillot, Olivier Chosidow

**Affiliations:** 1 Research Group Dynamyc, EA 7380, EnvA, Université Paris-Est (UPE), Maisons-Alfort & Créteil, France; 2 APHP, Hôpital Henri-Mondor, Department of Dermatology, UPEC, Créteil, France; 3 Department of Parasitology, College of Animal Science and Technology, University of Guangxi, Nanning, China; 4 Infections Diseases Department, Scabies Laboratory, QIMR Berghofer Medical Research Institute, Brisbane, Queensland, Australia; 5 Toxalim, INRA, INP-ENVT, INP-EI-Purpan, Université de Toulouse III Paul Sabatier, Toulouse, France; 6 Epidemiology and Infection Control Unit, University Hospital of Dijon, Dijon, France; 7 Cecaveto, Saint-Allouestre, France; 8 Department of Agriculture, Fisheries and Forestry, Queensland Animal Science Precinct, University of Queensland, Gatton Campus, Queensland, Australia; 9 Centre de Recherche BioMédicale (CRBM), EnvA, UPE, Maisons-Alfort, France; 10 APHP, Hôpital Henri-Mondor, Parasitology and Mycology, Department of Microbiology, DHU VIC, UPEC, Créteil, France; 11 Department of Parasitology and Mycology, Biopôle d'Alfort, Ecole nationale vétérinaire d'Alfort, UPE, Maisons-Alfort, France; 12 EA EpiDermE (Epidémiologie en Dermatologie et Evaluation des Thérapeutiques) and INSERM, CIC 1430, UPE, Créteil, France; University of California San Diego School of Medicine, UNITED STATES

## Abstract

**Background:**

Scabies is one of the commonest dermatological conditions globally; however it is a largely underexplored and truly neglected infectious disease. Foremost, improvement in the management of this public health burden is imperative. Current treatments with topical agents and/or oral ivermectin (IVM) are insufficient and drug resistance is emerging. Moxidectin (MOX), with more advantageous pharmacological profiles may be a promising alternative.

**Methodology/Principal Findings:**

Using a porcine scabies model, 12 pigs were randomly assigned to receive orally either MOX (0.3 mg/kg once), IVM (0.2 mg/kg twice) or no treatment. We evaluated treatment efficacies by assessing mite count, clinical lesions, pruritus and ELISA-determined anti-*S*. *scabiei* IgG antibodies reductions. Plasma and skin pharmacokinetic profiles were determined. At day 14 post-treatment, all four MOX-treated but only two IVM-treated pigs were mite-free. MOX efficacy was 100% and remained unchanged until study-end (D47), compared to 62% (range 26–100%) for IVM, with one IVM-treated pig remaining infected until D47. Clinical scabies lesions, pruritus and anti-*S*. *scabiei* IgG antibodies had completely disappeared in all MOX-treated but only 75% of IVM-treated pigs. MOX persisted ~9 times longer than IVM in plasma and skin, thereby covering the mite’s entire life cycle and enabling long-lasting efficacy.

**Conclusions/Significance:**

Our data demonstrate that oral single-dose MOX was more effective than two consecutive IVM-doses, supporting MOX as potential therapeutic approach for scabies.

## Author Summary

Scabies caused by the *Sarcoptes scabiei* mite affects many people worldwide and has been recently recognized by the WHO as a truly neglected tropical disease. Currently available treatments are insufficient to overcome this insidious disease and its co-morbidities for example impetigo, rheumatic heart disease and post-streptococcal glomerulonephritis. Treatment management is a major issue, as problems with compliance as well as mite resistance to current drugs are reported. Data have accumulated indicating that moxidectin could be a genuine new candidate drug for sustainable scabies control. To provide proof of concept, we utilized an experimental scabies pig model that closely resembles the human route of scabies infection. We demonstrated that a single moxidectin dose, when compared with the currently recommended two-doses ivermectin treatment routine, achieved a better and faster acaricidal efficacy. Importantly, the skin half-life of moxidectin is longer, potentially covering the entire mite life cycle. Our baseline data demonstrate in principle the potential and feasibility of moxidectin treatment for scabies, thereby enabling the move into larger high-powered efficacy and dose ranging studies in human populations. Moxidectin could indeed play a game-changing role in scabies control and has the potential to accelerate the steps towards elimination of this insidious disease.

## Introduction

Scabies is a very common dermatological condition, and a major public health burden globally [1,2]. Its recent addition to the WHO list of neglected tropical diseases highlights the urgent need of research to develop better scabies control [3]. Worldwide prevalence has been estimated between 100–130 million cases/year [4,5], and is increasing [6]. Scabies causes notable morbidity, and particularly affects the most vulnerable, e.g. young children, living in economically disadvantaged populations of tropical, and remote regions (e.g. Aboriginals in Northern Australia, Fiji islands) [5,7]. Scratching the skin disrupts the skin barrier, thereby providing an entry point for bacteria, which can become invasive, or cause post-infection complications (e.g. post-streptococcal glomerulonephritis, rheumatic heart disease) [8–10]. In economically advantaged settings, the risk of scabies outbreaks is particularly high in institutions (e.g. child and elderly care centers, or hospitals) [11,12], underprivileged populations [13], and immunocompromised individuals (e.g. HIV- or HTLV1-infected patients) [14,15].

The few available treatments for human scabies fail to control it. There are mostly topical agents like permethrin, benzyl benzoate, crotamiton, or malathion [16]. Ivermectin (IVM), is the only available oral drug [17], mostly used in institutional outbreaks [12], as mass drug administration in endemic areas [18], to treat severe, crusted scabies [19], or poorly compliant subjects. IVM, as well as topical permethrin have not been proven to have an ovicidal activity, and IVM shows a relatively short half-life. Therefore, multiple treatments with these drugs are generally required [20]. The situation is further complicated by the putative emergence of mites resistant to permethrin [21], and IVM [22,23], that might further spread [24] due to the increasing use of topical or oral IVM to treat head lice [25,26], or rosacea [27].

For the unmet need to treat scabies appropriately, moxidectin (MOX), another macrocyclic lactone, appears promising [28]. Compared to IVM, MOX stands out by its better pharmaco- and toxicokinetic profiles [29], mainly relating to its longer mean residence time in the body due to its high lipophilic nature [30–32]. Given the need to counteract the growing public health burden caused by scabies, we optimized the experimental porcine scabies model devised by Mounsey et al [33] and conducted a preclinical study to establish the efficacy, toxicity and pharmacokinetics of a single-dose MOX compared to the recommended two-dose IVM.

## Methods

### Experimental Porcine Model

#### Animals

**Fig 1:**
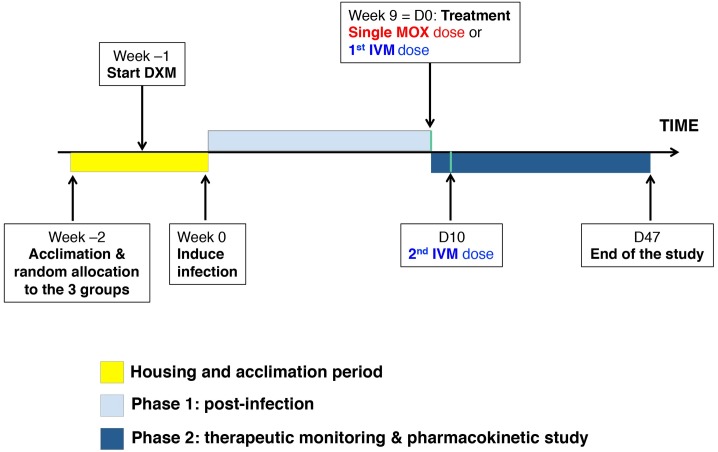
Diagram of the study design showing the 3 experimental phases: housing and acclimation phase, experimental phases 1 and 2. DXM, dexamethasone; D, day.

Twelve 3-week old *Sus scrofa domesticus* “Large white” breed, female siblings from the same pig farm (Gambais, France), were housed at Centre de Recherche Biomédicale (Maisons-Alfort Veterinary College, France). Their mean ± SD weight at arrival was 8.39 ± 0.86 kg. Pigs were initially mange-free and had never received any antiparasitic agents, including macrocyclic lactone compounds. All pigs were housed together 2 weeks post-arrival and before starting the study, to reduce stress and acclimatize them to the new environment (Fig 1). Drawing lots randomly assigned the pigs into three experimental groups (n = 4), housed separately in identical, experimental, climate-controlled units (temperature 21 ± 2°C, 50% ± 10% humidity, area 12 m^2^). Environmental enrichment included wood shavings on concrete floors that were cleaned daily. Feed was replenished once daily and tap water was available continuously. A 12-hour-light/12-hour-dark cycle was maintained (7-am on and 7-pm off). Each animal was physically examined by a veterinarian twice weekly to assure that animal-welfare standards were met. Invasive procedures (e.g. blood or skin samples) were kept to a minimum and performed under a short-term mild sedation to reduce stress or pain, using a mixture of 0.2 ml/kg Ketamine 1000 (Virbac, Carros, France), and 0.02 ml/kg Rompun 2% (Bayer Healthcare, Loos, France) given in a single intramuscular injection.

#### Ethics statement

The animals were handled in accordance with guidelines established by the French and European regulations for care and use of animals for scientific purposes (Articles R. 214–87 to 214–137 du Code Rural et de la Pêche Maritime, Décret 2013–118, and European Directive 2010/63/UE). The study was approved by our Institutional Animal Care and Use Committee, *Comité d’éthique pour l’expérimentation animale*, *Anses/EnvA/Université Paris-Est Créteil*, *France* (approval no: 02515.03). The ARRIVE Guidelines were used to design and report the study [34].

#### Infection of the pigs

The setup and maintenance of the experimental model was done in accordance with published procedures [33]. A synthetic glucocorticoid (dexamethasone base, Fagron SAS, Thiais, France) was started 1 week prior to infection, and pursued throughout the study at a daily oral dose of 0.2 mg/kg to promote the initial infection, and increase its intensity, and duration (Fig 1). Mite-infected skin crusts were collected from our first cohort of pigs infected with crusts from naturally infected pigs on a farm in Brittany (Saint-Allouestre, France). Crusts were dissected into small pieces (~0.5 cm^2^) containing between 600 and 800 mites, and inoculated directly into pigs’ ear canals. During the procedure, the pigs were mildly sedated to prevent crust expulsion by agitation, and ensure successful infection (S1 Video).

#### Study design

Following the initial experimental phases of immune-suppression and scabies-infection progression, treatments were administered on day 0 (D0), 9 weeks post-infection (Fig 1). MOX-treated pigs received 0.3 mg/kg once orally (oral paste for horses reformulated in non-enteric coated gelatin capsules Equest, Pfizer, Paris, France). IVM-treated pigs were given 0.2 mg/kg orally twice (on D0 and D10) (human formulation Stromectol, MSD France, Courbevoie, France). The MOX dose administered was equimolar to the IVM doses. Both drugs were given with food. Pigs were weighed on D0 to calculate the dose required; mean ± SD weight was 18.37 ± 2.6 kg. The infected control group remained untreated. Experimenters were blinded to the pigs’ treatment allocation. Drug efficacy and pharmacokinetics were determined from D0 to D47.

### Clinical Outcomes

#### Primary outcome

The primary outcome was the reduction of live-mite numbers counted in skin scrapings post-treatment with an endpoint being the complete absence of live mites on D14. The primary endpoint was planned to be evaluated on day 14, because the drugs used in this study have not be proven to have an ovicidal activity; and mites can continue to hatch after the drug administrations. Fourteen days were chosen to be in accordance with the life cycle of the mite [17]. Mites were collected and counted in skin scrapings, obtained at baseline D0 (just before treatment) and subsequently on D7, 14, 21, 28, 35, 42, and 47 post-treatment to estimate the percent treatment efficacy and percent mite-count reduction. An area of ~2 cm^2^ of each pig’s ear skin was scraped with a scalpel. Skin samples were examined in Petri dishes under a stereomicroscope (Nikon, SMZ645) and live mites present were counted. Mites were considered dead when no movement was seen after touching it with a needle and no gut movement was observed within 2 minutes.

#### Secondary outcomes

We designed a clinical score based on the skin area affected by scabies lesions, intensity of skin erythema, and crusting intensity. Every pig underwent weekly physical examination for post-infection scoring, and subsequently on D0, 7, 14, 21, 28, 35, 42, and 47 post-treatment, and photographs were taken.

To observe pruritus, every pig was monitored weekly for 15 minutes. Episodes of rubbing, and scratching were recorded. Flapping of the ears, rubbing against something, and scratching ears with a hind leg were considered pruritus (S2 Video). Scoring was done weekly post-infection, and subsequently on D0, 1, 5, 7, 14, 21, 28, 35, 42, and 47 post-treatment.

To monitor anti-*S*. *scabiei* var. *suis* IgG-antibody titers in sera, blood samples were collected once a week post-infection, and subsequently on D0, 7, 14, 21, 28, 35, 42, and 47 post-treatment. Pigs were mildly sedated, and blood was drawn by jugular vein puncture into untreated tubes. Sera were obtained by centrifuging for 10 minutes at 2,800 *g* and collecting the supernatant for storage at −80°C until assayed. A commercial enzyme-linked immunosorbent assay (Sarcoptes-ELISA 2001, AFOSA GmbH, Germany) was used according to the manufacturer’s validated instructions [35]. The microtitre plate was coated with a preparation of non-specific *Sarcoptes* antigens. Optical density values were read at 450 nm.

To monitor the impact of the drugs on hatchability, eggs were collected from skin scrapings on D0, 1, 7, and 21 post-treatment, placed in Petri dishes and incubated at 37°C, and 90% humidity until hatching.

### Moxidectin and Ivermectin Pharmacokinetics

Blood samples were collected in heparinized tubes (BD Vacutainer, BD, Plymouth, UK) on hours 1, 6 and 24 (on D0), and D2, 5, 7, 9, 12, 14, 22, 28, 36, 43, and 47 post-treatment. After centrifugation for 10 minutes at 2,000 *g*, plasma was obtained. Superficial skin biopsies were taken from each pig’s neck region on D2, 7, 9, 14, 22, 36, and 47 post-treatment with a standard 5-mm biopsy punch (KAI Europe, GmbH, Germany), extracting a sample of epidermis, and dermis. Hypodermal tissue if present was removed by dissection. Plasma and skin MOX and IVM concentrations were determined by high-performance liquid chromatography with fluorescence detection using a previously described and validated procedure [36]. Concentrations and linearities were similar (*r* = 0.99 over a 0.1–100 ng/ml concentration range), with limits of quantitation of 0.05 ng/mL and 0.1 ng/g. Pharmacokinetic parameter values were calculated using a non-compartmental analysis (Kinetica computer program version 4.2, InnaPhase, Philadelphia, PA). The area under the concentration-time curve (AUC_C-last_), and the mean residence time (MRT) were calculated from the time of first-administration to the time of last measurable concentration (T_last_), using the arithmetic trapezoidal rule. The peak concentration (C_max_), and time of C_max_ (T_max_) were read from the concentration vs. time plots for each pig, and T_1/2_ half-life was determined.

### Statistical analysis

The non-parametric Kruskal–Wallis H test was used to compare the groups on D0. The percent efficacy was calculated according to the following formula: Efficacy (%) = [(C–T)/C] × 100, where C is the mean number of live mites for the control group and T is the mean number of live mites for the treated group for each time point [37]. The percent reduction of the mite-count was calculated with the formula: Reduction (%) = [(Mpre–Mpost)/Mpre] × 100, where Mpre is the mean number of live mites on D0; and Mpost the mean number of live mites post-treatment on D7, 14, 21, 28, 35, 42, and 47 [37]. The mite-count and declines of clinical and pruritus scores over time within each pig group were tested for significance (*p* < 0.05) in a negative binomial regression model [38], with a robust variance estimate (zero-inflated negative binomial regression with Vuong’s test) [39] using STATA version12 software. ELISA results are expressed as relative optical density (%OD) values according to the formula: %OD = [(OD_sample_−OD_NC_)/(OD_PC_−OD_NC_)] × 100 where OD_sample_ means the OD of tested serum samples, OD_NC_ of negative control serum and OD_PC_ of positive-control serum. Pharmacokinetic parameter values for the different groups were compared with a non-parametric Mann–Whitney test, with *p* < 0.05 defining significance.

## Results

### Experimental Porcine Model

**Fig 2:**
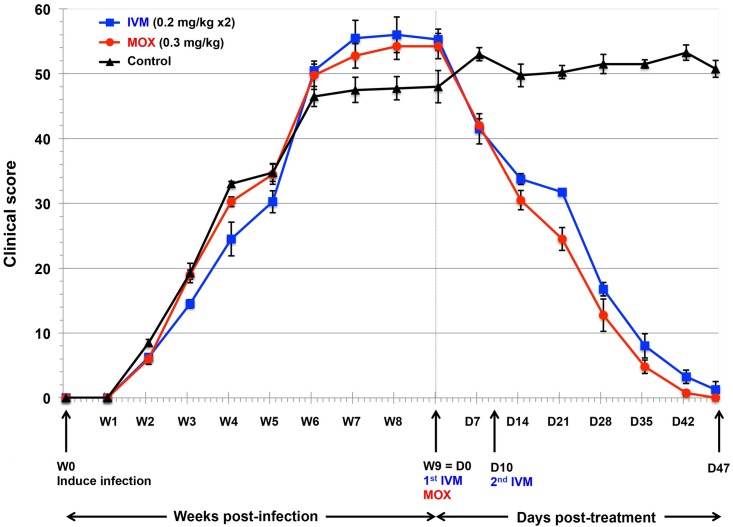
Clinical scores (mean ±SD) for the MOX- or IVM-treated and control pigs over time, from scabies-infection induction throughout the post-treatment period. Clinical scores are based on the scabies lesion-involved skin area (scored 0–6: 0, 0%; 1, <10%; 2, 10–29%; 3, 30–49%; 4, 50–69%; 5, 70–89%; 6, 90–100%), skin erythema (scored 0–4: 0, no erythema; 1, mild; 2, moderate; 3, severe; 4, extremely severe) and crusting intensity (scored 2× 0–4: 0, no crust; 1, grey to white, thin and irregular 1–2 mm crust; 2, 2–5 mm crust; 3, grey-brown >5 mm crust; and 4, >5 mm, hard crust). The score was calculated for 5 different anatomic sites and added. W, week; D, day.

**Fig 3:**
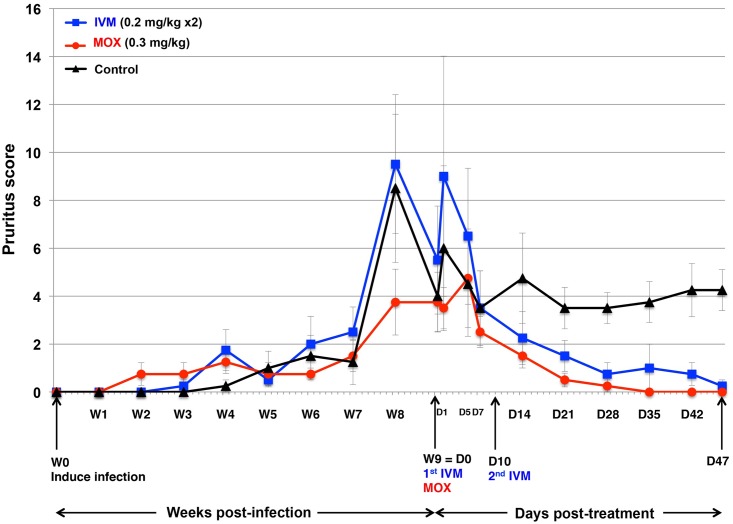
Pruritus scores (mean ±SD) over time from scabies-infection induction throughout the post-treatment period for MOX- and IVM-treated groups and control pigs. W, week; D, day.

**Fig 4:**
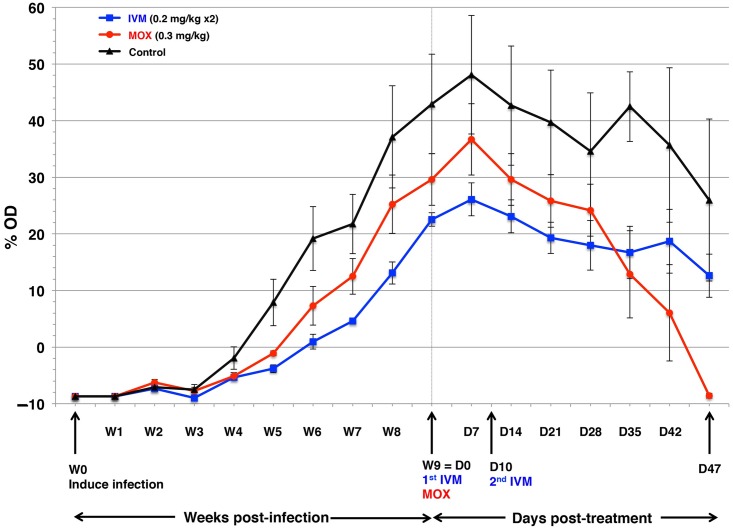
ELISA-assessed serological responses (expressed as mean %OD ± SD) after *S*. *scabiei* infection throughout the post-treatment period of the MOX- and IVM-treated groups and control pigs. W, week; D, day.

After infection, the first cutaneous lesions were visible in the ears and then spread to the entire body. Crusts appeared at 4 weeks post-infection. Accordingly, clinical scores (Fig 2), pruritus scores (Fig 3), and anti-*S*. *scabiei* IgG-antibody serum titers (Fig 4) rose steadily post-infection. Scabies treatment commenced at D0, when mite counts had risen above 100 mites per scraping (Table 1) and all pigs showed clinical signs of mite infection associated with intense itching. At baseline (D0), the 3 groups were comparable in terms of mite counts (*p* = 0.703), clinical scores (*p* = 0.146), pruritus scores (*p* = 0.878), and IgG antibody levels (*p* = 0.166). No clinical signs of intolerance to IVM, or MOX were observed during the 47-day observation period. Dexamethasone side effects were mild (enhanced appetite and hairiness), as previously reported [33].

**Table 1:** Primary outcome of MOX, IVM or no treatment of experimental scabies in pigs.

Study Day	MOX (*n* = 4)	IVM (*n* = 4)	Controls (*n* = 4)
0			
No. of mites per scraping (mean ± SD)	111 (27.8 ± 28.7)	108 (27 ± 26.6)	115 (28.8 ± 40.8)
Count range, *n*	5–69	3–61	3–89
7			
No. of mites per scraping (mean ± SD)	3 (0.8 ± 1.5)	89 (22.3 ± 39.2)	169 (42.3 ± 72.5)
Count range, *n*	0–3	1–81	3–151
Reduction, % (range)	97% (96–100%)	18% (NA–78%)	
Efficacy, % (range)	98% (93–100%)	47% (46–97%)	
14			
No. of mites per scraping (mean ± SD)	0	32 (8 ± 15.3)	84 (21 ± 19.5)
Count range, *n*	0–0	0–31	2–42
Reduction, % (range)	100%	70% (49–100%)	
Efficacy, % (range)	100%	62% (26–100%)	
21			
No. of mites per scraping (mean ± SD)	0	2 (0.5 ± 1)	144 (36 ± 37.1)
Count range, *n*	0–0	0–2	3–76
Reduction, % (range)	100%	98% (97–100%)	
Efficacy, % (range)	100%	99% (94–100%)	
28			
No. of mites per scraping (mean ± SD)	0	47 (11.8 ± 23.5)	139 (34.8 ± 39.7)
Count range, *n*	0–0	0–47	2–84
Reduction, % (range)	100%	57% (23–100%)	
Efficacy, % (range)	100%	66% (44–100%)	
35			
No. of mites per scraping (mean ± SD)	0	37 (9.3 ± 18.5)	119 (29.8 ± 33.2)
Count range, *n*	0–0	0–37	10–79
Reduction, % (range)	100%	66% (39–100%)	
Efficacy, % (range)	100%	69% (53–100%)	
42			
No. of mites per scraping (mean ± SD)	0	27 (6.8 ± 13.5)	75 (18.8 ± 21.5)
Count range, *n*	0–0	0–27	2–49
Reduction, % (range)	100%	75% (56–100%)	
Efficacy, % (range)	100%	64% (45–100%)	
47			
No. of mites (per scraping (mean ± SD)	0	13 (3.3 ± 6.5)	182 (45.5 ± 52.2)
Count range, *n*	0–0	0–13	1–104
Reduction, % (range)	100%	88% (79–100%)	
Efficacy, % (range)	100%	93% (45–100%)	

SD, standard deviation; NA, not applicable

### Clinical Outcomes

#### Primary outcome

Post-treatment, the mite-count declined significantly faster in the MOX-treated pigs compared to those treated with IVM (*p* = 0.001) (Table 1). At D14, all four MOX-treated pigs but only two of the four IVM-treated pigs were mite-free. MOX efficacy was 100% compared to 62% (range 26–100%) for IVM, with respective mite-count reductions of 100% and 70% (range 49–100%) (Table 1). From D14 onwards, no mites were observed in the scrapings from the MOX-treated pigs. Among the IVM-treated pigs, one remained infected with live mites observed continuously until D47. In all untreated control pigs the mite counts remained high.

#### Secondary outcomes

Post-treatment, the mean clinical scores for MOX and IVM groups differed significantly from each other (*p* = 0.021), and from those of the controls (*p* = 0.0001) (Fig 2). By D47, clinical scabies lesions had disappeared completely in all the MOX-treated pigs and in three of the IVM-treated pigs, while the lesion levels in the controls remained high.

Immediately post-treatment (D1), pruritus increased in both treated groups, followed by a decline (Fig 3). The mean pruritus scores of MOX-treated group were lower but did not differ significantly compared to the IVM-treated group (*p* = 0.239), however differed significantly from those of the controls (*p* = 0.0001).

Post-treatment, mean antibody titers in MOX-treated pigs decreased markedly but remained constant in IVM-treated group (Fig 4). Mean antibody titers were constant and high in the controls.

At D0, 10 eggs from each group were incubated and all (except one from the control group) hatched. On D1, nine out of nine, eight out of ten and seven out of seven eggs, respectively, from the MOX-, IVM-treated and control groups hatched. On D7 and D21, no eggs were present in scrapings from MOX-treated pigs. Four (D7) and two eggs (D21) were obtained from the IVM-treated pigs, and all of them hatched.

### Moxidectin and Ivermectin Pharmacokinetics

#### Plasma pharmacokinetics

**Fig 5:**
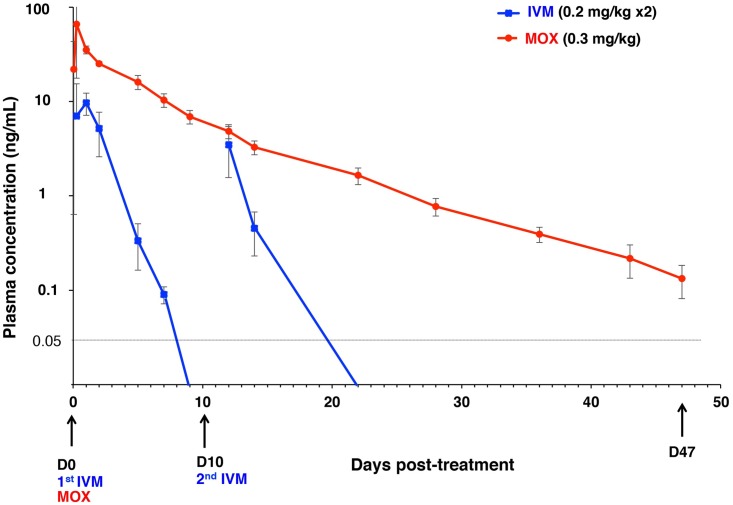
Plasma MOX and IVM concentrations (mean ± SD, ng/ml) after oral intake in scabies-infected pigs. Concentrations measured on hour 1, 6 and 24 of D0 for MOX and hour 6 and 24 of D0 for IVM and on D2, 5, 7, 9, 12, 14, 22, 28, 36, 43, and 47 post-treatment are depicted. W, week; D, day.

Drugs were detected in plasma within 1 hour post-administration in all MOX-treated pigs, and in three IVM-treated pigs. MOX was detectable until the study-end but IVM only until 7–9 or 12 days post-first, or second administration. About half a day post-administration, MOX achieved a ~6 times higher plasma C_max_ than IVM (Table 2 and Fig 5). Administration of a second IVM-dose on D10 did not raise IVM levels above MOX levels. The AUC_C-last_ was ~12 times larger for MOX than for IVM, reflecting a much better exposure of the entire organism to MOX than to IVM. The greater persistence of MOX in plasma was reflected by a longer elimination T_1/2_ and MRT (Table 2).

**Table 2:** MOX and IVM pharmacokinetic parameters of scabies-infected pig plasma and skin.

Site	C_max_	T_max_ (day)	AUC_C-last_	T_1/2_ (day)	MRT (day)	T_last_ (day)†
Plasma	(ng/ml)		(ng.day/ml)			
MOX	70.1 ± 42.3	0.5 ± 0.3	237.5 ± 41.9	7.2 ± 1.1	7.1 ± 0.9	47
IVM 1^st^ dose	12.6 ± 3.9*	0.6 ± 0.4	19.8 ± 3.3*	0.8 ± 0.1*	1.6 ± 0.3*	7–9
IVM 2^nd^ dose	3.5 ± 1.9*	12 ± 0	8.2 ± 4.4*	–	–	14–22
Skin	(ng/g)		(ng.day/g)			
MOX	602.7 ± 68.2	2.0	5294.6 ± 1280.4	8.6 ± 2.8	11.8 ± 2.0	47
IVM 1^st^ dose	54.8 ± 19.2*	2.0	123.3 ± 33.3*	1.0 ± 0.2*	2.7 ± 0.3*	9–14
IVM 2^nd^ dose	–	–	–	–	–	14–22

MOX and IVM parameter values are expressed as mean ± SD after oral intake.

–, could not be determined.

*p < 0.001 vs. MOX (compared with a non-parametric Mann–Whitney test).

^†^, day count from the time of first-administration to the time of the last measurable concentration.

#### Skin pharmacokinetics

**Fig 6:**
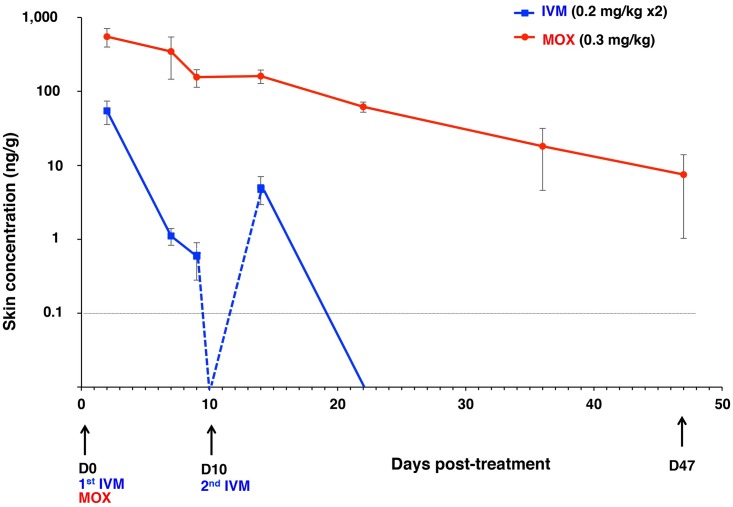
Skin MOX and IVM concentrations (mean ± SD, ng/g) after oral intake in scabies-infected pigs. Concentrations measured on D2, 7, 9, 14, 22, 36, and 47 post-treatment are depicted. Predicted parts of the curves are indicated as a dashed line. W, week; D, day.

Both drugs had reached the skin at D2. MOX was extensively distributed within the skin with MOX skin C_max_ values being ~10 times higher than those measured in plasma (Table 2 and Fig 6). This is also reflected by the very high AUC_C-last_ values. Skin MOX T_1/2_, and MRT were longer than those in plasma. Compared to MOX much less IVM accumulated in the skin and the drug could not be detected in 3 of 4 pigs beyond 9 and 12 days post-first, and second administration respectively. Skin IVM T_1/2_, and MRT were longer than those in plasma but much shorter than respective MOX values.

## Discussion

Given the rising concern about scabies worldwide, and the emergence of drug-resistance in mites, an immediate alternative therapeutic option is an imperative. In this preclinical study, performed in an acknowledged animal model, single-dose MOX treatment (0.3 mg/kg) was more efficient at killing mites, reducing the immune response, and possibly inflammation than two IVM doses (0.2 mg/kg each).

The single MOX-administration resulted in rapid, and notable acaricide efficacy of 98% (range 98–100%) seven days post-treatment, and 100% from D14 onwards. This was not the case for IVM-treated pigs, with half of the group not cured on D14, the primary study endpoint, and one of the four pigs being still infected at study-end. Clinical scores demonstrated significantly different recovery rates between MOX, and IVM, or controls. On D47, no cutaneous lesions were seen on any of the MOX-treated pigs but only on three of the four given IVM. Also, the MOX-treated pigs recovered faster than the IVM-treated animals. After D1, pruritus scores declined sharply for both treated groups, in accordance with literature [40], but more markedly for MOX-treated pigs, albeit not statistically different. Pruritus persisted for several days post-treatments, in accordance with observations made on humans with the so-called “post-scabies syndrome” [16]. Surprisingly, scores rose similarly for the controls, perhaps attributable to imitative behavior [41]. The pruritus assessment was limited as a number of factors could have influenced the pigs’ itch, e.g. presence of observers in the room, time of the day, room temperature, humidity [42]. Video recordings could improve the monitoring of scratching behavior [40]. The serological responses throughout infection and post-treatment were similar to those reported by Kessler et al [35]. Despite the small animal number, which is the main limitation of our trial, the analyses of primary and secondary outcomes highlighted that all results converged to demonstrate that MOX was more effective than IVM at every study-time point. The primary endpoint was relevant and the analyses attested that our cohort size (even if small) was reliably representative.

The excellent MOX efficacy could be directly reflected by its better pharmacokinetic characteristics: rapid absorption, larger distribution and remarkably longer persistence in plasma, and importantly, also in the skin. MOX remained detectable in plasma, and the skin for the entire 47 days of observation post-administration, while IVM was undetectable 7–9, and 12 days post-administrations. Our data are comparable to those from studies on pigs of similar age, and body weight [43,44], other animal species [30,45,46–48], and humans [49,50]. Herein MOX concentrations measured in the skin were considerably higher than those found in plasma, in agreement with Lifschitz et al [32], thereby confirming that the skin represents a key reservoir for MOX storage, perhaps because of its high lipophilicity and lower susceptibility to efflux via ABC transporters [29,51].

The dexamethasone use is fundamental in this model to standardize infection rates. It can’t be ruled out that dexamethasone interfered with MOX, and IVM pharmacokinetics as dexamethasone is a P-glycoprotein substrate, and a negative interaction might occur when dexamethasone, and macrocyclic lactones are given simultaneously [52,53].

The main study strength was the robustness, and the trustworthiness of the experimental model. Pigs have been recognized as a preferred model to investigate skin disease in translational dermatological research [54], as the porcine epidermal structure is very similar to humans [55], and both immune systems share marked similarities [56]. In addition, human infectious disease experiments in pigs are considered to be very predictive of therapeutic treatments in humans [57], including pharmacokinetics, and epidermal drug absorption [58]. In this model, pigs developed clinical manifestations closely resembling human scabies [59]. Our observations successfully replicated the Australian reports [33,60] for scabies development, e.g. timescale for lesion (erythema and crusts) appearance, pruritus, and infection severity. In addition, the model was optimized for this therapeutic trial, and standardized infections in all pigs by weighing out the inoculating samples. We created a new scoring system to clinically monitor cutaneous lesions, and pruritus.

With regards to the increasingly difficult treatment options for scabies, a number of advantageous characteristics have been highlighted for MOX in this trial. Firstly, a major point, and an increasing concern, arising from this trial along with other studies [28,61,62], is the documentation of IVM failure. The poorer IVM efficacy observed might be explained by its shorter half-life, and lower penetration into hyperkeratotic areas of skin (or skin with less fat), which may lead to a suboptimal mite exposure to IVM. More pharmacokinetic studies are needed to assess the drug quantity in the different skin layers, and in crusted skin tissues. Furthermore, the mechanisms of epidermal drug transport in particular in the stratum corneum, remains unclear [63]. Secondly, two consecutive publications reported mites resistant to IVM treatment [22,23]. Even though MOX acts on the same receptor as IVM, cross-resistance is not certain. To date resistance to IVM is much more widespread than to MOX in various nematodes [29], and MOX is still more effective than IVM against IVM-resistant parasites [64]. Thirdly, no adverse events occurred in response to MOX treatment, in line with other studies indicating that MOX may be less toxic than IVM because MOX is a poorer substrate for P-glycoproteins than IVM [29,65]. MOX had a wider safety margin when given to Collie dogs, which are known to have a multidrug-resistance protein-1 gene (*MDR1*)-deficient genotype, rendering them hypersensitive to the P-glycoprotein substrate [66]. In the first phase I study evaluating MOX administration to healthy human volunteers [50], MOX was well-tolerated for a dose range of 3–36 mg, further confirmed by Korth-Bradley et al [67]. Lastly, we showed here for the first time, that eggs are not harmed by either drug. Nonetheless, newly hatched mites may be killed by the long-lasting MOX persisting in the skin, leading to prolonged MOX efficacy. We propose that the drug’s metabolic stability in the host-tissue may indeed cover the entire 14-day mite life cycle, and a single-dose MOX might suffice to clear infection. Resource-poor communities are known to have poor compliance to topical regimens [68], due to a shortage of medical advice, nursing assistance, and because of the discomfort using a cream in tropical settings, and on irritated, or superinfected skin. Likewise, topical agents are difficult to use in care facilities for the elderly, mentally-ill, or disabled patients where outbreaks occur frequently. The major advantage of a single dose might increase compliance, and be a key determinant of treatment success, in contrast to the two IVM-administrations. This could also be a highly relevant option in outbreaks, to prevent immediate reinfestation.

For many years, IVM has been used to treat onchocerciasis, donated free-of-charge by the philanthropic Mectizan Donation Program [69]. Results of recent phase II, and III human clinical trials [70,71] demonstrated that MOX is likely more effective than IVM at controlling onchocerciasis, and has the potential to advance efforts to eliminate this disease. With that objective, MOX is presently being developed as an alternative treatment for onchocerciasis, and being considered for regulatory authority-submission for human use [72]. The limited treatment options currently available for scabies may require us to pursue this path promptly. Further studies should investigate the efficacy and dose selection of MOX in humans, indeed a pivotal Phase II trial for MOX for scabies elimination has now been funded and will soon commence [73].

## Supporting Information

S1 Video Infection procedure. The video shows the infection procedure. Mite-infected skin crusts are directly introduce deep into the pigs’ ear canals. Pigs are mildly sedated during the procedure.
(MOV)

S2 Video Pruritus behaviour. The video shows pruritus behaviour. The pigs is flapping of the ears and scratching ears with his hind leg.
(MOV)
